# Practical guidance on publishing plain-language summaries for scientific manuscripts with an Asia-Pacific audience

**DOI:** 10.1186/s13104-025-07433-7

**Published:** 2025-09-02

**Authors:** Blair Hesp, Bert Yu-Hung Chen, Shobana Ganesan, Joyce Hiu Yan Lee, Sharon Wei Ling Lee, Anna Onuma, Shu Wang, Alvin Wei, Julie Yuan, Tim Stentiford

**Affiliations:** 1 First In Human, Kainic Medical Communications Ltd, Dunedin, New Zealand; 2 Vercentrys Taiwan, Taipei, Taiwan; 3 Cactus Life Sciences Pvt Ltd (part of Cactus Communications), Mumbai, India; 4 Nucleus Global, an Inizio Company, Sydney, Australia; 5 Costello Medical Singapore Pte Ltd, Singapore, Singapore; 6 Lilly Suzhou Pharmaceutical Co. Ltd, Shanghai, China; 7 L’Oréal Research and Innovation, Shanghai, China; 8 John Wiley & Sons Singapore Pte Ltd, Singapore, Singapore

**Keywords:** Accessibility, Asia-Pacific, Communication, Plain language summary

## Abstract

Plain language summaries (PLSs) of scientific publications are intended to distill full-length scientific publications into a short, inclusive, accessible format that can be rapidly comprehended by all potential readers, but often retain cultural and linguistic barriers to accessibility, particularly for audiences in the Asia-Pacific region (APAC). Various publications have attempted to provide guidance on how to prepare PLSs, but there have been limited attempts to standardize structure or content and there is no universal guidance for PLSs. Furthermore, PLSs pertaining to research in patient populations in APAC, or relating to conditions that are prevalent in APAC, should be developed using language and imagery appropriate for the intended audience. This commentary aims to outline key considerations for preparing a PLS for an APAC audience, including language, imagery, and font selection. Developing culturally-sensitive PLSs for potential readers in APAC may increase the audience for the scientific literature and assist effective engagement in the region.

## Introduction

Scientific publications are generally lengthy and follow a standard format intended for an expert audience. There is a presumption that expert readers will have adequate time to study and deeply contemplate the nuance of a publication beyond the topline results.

Plain language summaries (PLSs) of scientific publications distill scientific publications into a short, inclusive, accessible format that can be easily comprehended by any potential reader [1]. However, many PLSs retain technical language and offer an inadequate explanation of the results [2, 3]. Even the term ‘PLS’ has become mired in technicality with competing definitions [4, 5].

The Good Publication Practice 2022 (GPP 2022) guidelines for publishing industry-funded research offered a broad definition of a PLS encompassing written lay summaries, videos, podcasts, graphical abstracts or infographics [6], although the general public may consider a PLS to be a short, lay version of a scientific publication, written to engage a non-expert reader by using ‘plain English’. This commentary uses the broad definition of PLSs provided in the GPP 2022 guidelines.

Most scientific manuscripts intended for an international audience are published in English, which can be a barrier to accessibility [5]. The ubiquity of the English language across scientific publications has consequently resulted in predominantly Western cultural imagery being applied to content, including PLSs [7, 8]. Furthermore, most research into communication in healthcare settings has occurred in English language-dominant settings, or has focused on the challenges of attempting to apply Western models of communication in Asian contexts [9].

Although discussion regarding PLSs has focused on extending the reach of scientific publications to non-expert groups, such as patients, patient organizations, and caregivers, consideration of the diversity within each group has been limited [10]. This approach also takes a Western view of the purpose of PLSs being to disseminate data to patients and facilitate engagement [10], rather than make data accessible to an audience who is less familiar with the topic or for whom English is not their first language. In particular, PLSs offer a succinct summary that can be easily translated using online tools that can engage healthcare providers for whom English-language manuscripts are inaccessible [11]. However, the need to develop effective, culturally sensitive methods of communicating with patients in the Asia-Pacific (APAC) region to enhance shared decision-making and person-centered care have been noted [9, 12].

PLSs pertaining to research in patient populations in APAC, or conditions prevalent in APAC, should be developed using language and imagery that is appropriate for the intended audience [4, 13, 14]. Effective communication of data in APAC may influence treatment approval and reimbursement, and affect how the general public perceives diseases and conditions, whether patients seek treatment, and how healthcare providers select treatment. Therefore, there have been calls to create more culturally relevant content for audiences whose first language is not English [8, 9], moving beyond the suggestion that simply translating English-language content into another language facilitates cross-cultural dissemination [10, 11]. There has also been a call for action towards greater intercultural understanding of how to communicate medical data in Asian countries [9, 11].

For example, Stoll et al. (2022) [4] described PLSs as a “research translation service”, highlighting that PLSs should be readily accessible across geographies and skill levels. This approach may assist speakers of English as a second-language in understanding research outcomes reported in scientific manuscripts. Accordingly, there has been an increasing prevalence of PLSs among journals published in APAC [15].

This commentary aims to outline key considerations that should be applied when preparing a PLS for an APAC audience. PLS publications, which are distinct from PLSs, are outside the scope of this commentary.

## Principles of PLS Preparation in APAC

Several key underlying principles have been developed to help guide authors preparing PLSs that target an APAC audience (Table).

**Table:** Key recommendations for preparing a PLS relating to research in an APAC population or with a predominantly APAC audience

PLS consideration	Recommendation
Structure	• Include the title, first author's family name, and citation details, to aid discovery of the parent publication [3,17] • Use figures that can be universally understood [19] • Incorporate a conclusion statement that provides an overarching single message [19–21]
Language selection	• Consider the nuances of the English language • Avoid terminology that may be ambiguous or have a dual meaning • Common English-language metaphors and cliches should also be avoided
Font selection	• Use a standard sans serif font, such as Arial or Calibri • Avoid using red font for written content
Numbers	• Avoid highlighting the number 4 because of its association with death
Image selection	• Imagery should reflect the relevant study population or audience [12] • Authors should be conscious of areas where cultural symbology may differ between Western and APAC cultures • Care should also be taken to avoid cultural appropriation of imagery
Editorial support	• When editorial support has been provided to the authors, or the PLS has been developed by a third party (e.g., an internal team at the publisher), this should be transparently acknowledged

APAC, Asia-Pacific region; PLS, plain-language summary

### Structure

Various recommendations on preparing PLSs have been published, but attempts to standardize structure or content have been limited [15]. There is currently no universal guidance for PLSs [10], but minimum standards have been proposed [16]. To our knowledge, no guidance has been developed to accommodate the diverse cultural and linguistic communication norms of APAC audiences.

PLSs should include the title and first author’s family name, and ideally citation details, to aid discovery of the full parent publication [3, 17, 18]. Where possible, figures should communicate results because figures can be universally understood – a ‘wall of text’ is a significant barrier to engagement and understanding for many readers [19]. An overarching conclusion statement, providing a single takeaway message from the publication can guide interpretation of the data [19–21]. Authors are also encouraged to include important limitations of the study that provide relevant context in the conclusion statement [13, 21]. Providing detailed study methodology is of lower priority in PLSs, but if included, should be presented visually [18, 21, 22].

Although reading may follow a top-to-bottom and right-to-left pattern in some Asian languages, the layout of PLSs should follow the English-language format of top-to-bottom and left-to-right for consistency between the full manuscript and its accompanying PLS [19].

### Language selection

Caution should be exercised when attempting to write in ‘plain English’ with a relatively low reading age [10]. For example, language that may be classified as suitable for persons with an English-language reading age equivalent to a 12-year-old native speaker may not be as easily understood by speakers of English as a second language [10, 22]. Using technical terms with an accompanying explanation can also aid understanding of potentially novel terminology and the underlying manuscript [18, 22].

There is a risk that some words reflect the dialect of English spoken by the Author(s) or have dual meanings, making interpretation challenging. For example, ‘difficult’ should be used instead of ‘hard’ to describe challenging content. Common English-language metaphors and cliches should also be avoided because the intended meaning may not be easily understood or translated into other languages and cultures.

Nuances of the English language, which appear inconsistent with common grammatical rules, can also be difficult to interpret. For example, the word ‘holistic’ could be easily associated with religion by a speaker of English as a second language because of its proximity to ‘holy’ and ‘holiness’ rather than ‘whole’, given the differences in spelling. Words such as ‘invaluable’ can also use the negative prefix ‘in-’, but the writer’s intent is to reinforce positive value.

There should also be consideration of the customary use of American versus British English in different APAC countries. British English may be the default in former British colonies where English is widely spoken, such as India, Pakistan, Singapore, Australia and New Zealand, whereas American English is more commonly used in Japan, Korea, The Philippines and other countries where English is predominantly learned as a second language and exposure occurs via an American lens. American pronunciation and accents may also be encouraged in podcasts and video content because this may be more familiar to speakers of English as a second language. Subtitles and/or transcripts should be provided for video and audio PLSs, including in the local language(s) of the target audience in addition to English.

### Numbers

While the number 8 is associated with happiness and luck in some APAC cultures, the number 4 can be associated with death. The number 9 may also be associated with ‘suffer’ in Japan. Dates should also be spelt out to avoid confusion between MM/DD/YYYY and DD/MM/YYYY formats.

### Font selection

Standard, commonly used, sans serif fonts, including Arial or Calibri, can aid readability [21]. A preference in font style for certain letters has also been anecdotally observed in APAC (e.g.,’ and ‘’ are preferable to “” and “”).

Thought should also be given to font color [21]. Red font should be avoided in the context of medical literature. It is considered taboo to write a living person’s name in red ink in many Asian countries, especially if this was done by healthcare providers in the presence of patients. Writing names in red is associated with imminent death in some mythologies and diminishes the common association of the color red with happiness.

Font colors should also strongly contrast against the background. For video subtitles, white font with a black outline or background maximizes contrast and readability.

### Imagery

Imagery should reflect the relevant study population or audience [10, 18]. For example, the ethnicity of people in images should be consistent with the ethnicity of the study or target population. Care should be taken to avoid applying Western cultural stereotypes of Asian populations [23]. Likewise, the plurality of ethnicities and cultures within APAC should be recognized [9]. If multiple ethnicities are represented, simple imagery with neutral color tones and/or clothing should be chosen. There should be a balanced representation of gender and age, where relevant.

Authors should be conscious of areas where cultural symbolism may differ between Western and APAC cultures. For example, an owl is considered a symbol of wisdom in the West, but is associated with death in Māori culture in New Zealand.

Images of tattooed people should also be avoided in Japan and images of people who are deceased may be taboo in some cultures, such as the Australian Aboriginal and Torres Strait Islander community [24]. Appropriate warnings may need to be included before presenting imagery of the deceased or naming the deceased in written text [24].

Care should also be taken to avoid cultural appropriation of imagery, especially when using artificial intelligence to assist in generating images. For example, the use of traditional imagery (e.g., the haka or moko [traditional facial tattoos]) by non-Māori in any setting, but especially overseas, may be considered offensive by New Zealanders.

Imagery should additionally be limited to what is essential for conveying key messages in a clear and meaningful manner. Complex images should be accompanied by a simple written explanation of the intended message [18]. This avoids unnecessary distractions from the main narrative of the PLS.

### Editorial support

PLSs should ideally be written by the authors themselves [13], but this may not always be feasible. Authors are encouraged to familiarize themselves with both journal guidelines and independent recommendations on how to prepare a PLS; data suggests that some authors may not understand that a PLS is meant to stand alone as a novel, complementary output that sits alongside a full manuscript [21].

When non-author editorial support has been provided to the authors developing a PLS (e.g., a professional medical writer or an internal team at the journal publisher), this should be transparently acknowledged. Acknowledgment of support would ideally occur within the PLS itself, but should also be included within the acknowledgements section of the manuscript. Any use of artificial intelligence to support the development of either the PLS text or imagery should also be disclosed [25].

### Other considerations

When developing a PLS reporting data from an APAC population or for an APAC audience, consulting with colleagues in the region is recommended [10, 18, 19, 21]. Likewise, authors and other stakeholders (e.g., study sponsors) involved in developing scientific manuscripts in APAC are encouraged to prioritize PLS development, beyond their typical focus on the main manuscript. Research has shown that PLSs can help readers gain an accurate understanding of the main manuscript, and subsequently increase interest in accessing the full text, among other benefits [26–28]. This highlights the value of the PLS as a communications tool that can remove language barriers to increase awareness of critical research among stakeholders.

## Limitations of guidance of PLS development in APAC

No guidance has been provided for PLS format selection, which may be limited by journal selection and resource availability. However, authors are encouraged to advocate for the use of PLSs alongside scientific manuscripts and consider providing a PLS as a supplementary appendix, if not formally supported by their journal of choice.

Data regarding optimal PLS format are conflicting. A written lay summary or video may achieve the greatest comprehension, understanding and enjoyment, although these data were collected from a largely American, native English-speaking population [29]. Written PLSs are also easier to analyze for complexity and comprehension than more visual formats, such as infographics and graphical abstracts, because analysis can be automated. Other data from a UK-based, native English-speaking population suggests that readers prefer infographics over written text [30]. Difficulty in understanding audio-based PLSs resulting from speaker accent, speech speed, or English dialect, which could be barriers to comprehension for speakers of English as a second language, have not been prospectively investigated.

Overall, there is limited empirical research available to inform PLS development recommendations [4, 9]. This highlights the need for further investigation into the effectiveness of PLSs, and how they are comprehended by readers outside of predominantly English-speaking countries [9].

## Outlook

PLSs offer an opportunity to create a short summary of a scientific manuscript that increases the accessibility of technical content. Developing culturally-sensitive PLSs for APAC audiences may increase engagement with the scientific literature in the region.

## Data Availability

No datasets were generated or analyzed during the current study.

## Abbreviations

APAC: Asia-Pacific region
GPP 2022: Good Publication Practice 2022
PLS: Plain-language summary
